# Automatic design and optimization of educational space for autistic children based on deep neural network and affordance theory

**DOI:** 10.7717/peerj-cs.1303

**Published:** 2023-03-28

**Authors:** Linya Chu, Min Lee

**Affiliations:** 1 Department of Spatial Culture Design, Graduate School of Techno Design, Kookmin University, Seoul, Korea; 2 Graduate School of Techno Design, Kookmin University, Seoul, Korea

**Keywords:** Autistic children, Affordance theory, Educational space optimization design, The layout recommendation algorithm

## Abstract

In recent years, the incidence of autistic children has shown rapid growth worldwide. The rapid development of education and rehabilitation institutions for autistic children is of great significance to the rehabilitation of this group. However, the research on indoor space environments and functional facilities for autistic children in China is still in its infancy. Reasonably and effectively, zoning the education and rehabilitation space for autistic children can promote better communication and learning between autistic children and rehabilitation therapists and effectively promote the rehabilitation progress of autistic children. However, the existing education and rehabilitation space for autistic children has some problems, such as unscientific indoor partition, indoor space layouts mainly relying on manual work, heavy workload and low efficiency. Therefore, it is of great research value and practical significance to explore the intuitive design and optimization of the education and rehabilitation space layout for autistic children. This study first evaluates and optimizes the educational space for autistic children based on the affordability theory. Then, this study proposes a layout recommendation algorithm based on deep learning, which is used to improve the layout efficiency of the education and rehabilitation space for autistic children and realize real-time online layout. The scene information is digitized in binary code. The segmentation and layout network models are constructed through bidirectional long short-term memory (LSTM) to discover the long segment pre-segmentation of house type and obtain the layout results. The word embedding algorithm is used to abstract the cross features between each vector segment, and the dimension of the feature matrix is reduced to improve the speed and accuracy of the layout scheme recommendation. The experimental results show that our method can learn the design rules from the data set and has achieved better results than the existing methods. This study provides an adequate theoretical basis and design reference for the research of residential education space for autistic children.

## Introduction

In recent years, childhood autism has been increasing (Lemonnier et al., 2012). The Report on the Development of China’s Autism Education and Rehabilitation Industry shows that there are more than 10 million autistic patients and more than two million children in China, with an annual growth rate of nearly 200,000. Childhood autism has ranked first as a mental disability in China (Wucailu Children’s Behavior Correction Center, 2015). At present, autism rehabilitation centres have been built in Beijing, Nanjing, Qingdao, Guangzhou, Chongqing and other cities in China, but 83.3% of them are private institutions. The vast majority of rehabilitation spaces and environments are poor, and rehabilitation equipment is incomplete. Desideri et al. (2018) believes that autism, a specific disease, requires a long rehabilitation cycle. Good educational spaces and professional rehabilitation training can enable autistic children to recover various functions of their bodies as soon as possible (Desideri et al., 2018). Therefore, in the context of the increasing number of autistic children and the growing attention paid by all sectors of society to this particular group, it is very worthwhile for experts and scholars in the interior design field to seriously study the improvement of the service system for autistic children and the optimization of the rehabilitation space design for autistic children. Mazurek et al. (2020) believes that the research on the education and rehabilitation space of autistic children is a complex systematic project involving rehabilitation medicine, child psychology, pedagogy, design, ergonomics and other disciplines. Designers should start from the rehabilitation needs of autistic children, fully respect the psychological characteristics of autistic children, and understand the behavior of patients (Mazurek et al., 2020). Qi, Han & Yin (2021) believe it is necessary to design an educational rehabilitation platform for children with autism.

The term “affordability” was put forward by Gibson (1977), a famous American ecological psychologist, in 1977 to explain the relationship between organisms and substances and environmental configurations. Since the concept of “affordability” was created to this day, it has gradually aroused extensive interest in various fields because it provides innovative thinking for cognitive psychology (Norman, 2004). The theory of affordability has been applied in many areas, such as psychology, pedagogy, ecology, design, *etc*. It provides a new perspective for designing and improving the educational space environment for autistic children. Kelly (2018) believes that in creating education and rehabilitation space for autistic children, designers should try their best to provide an activity space to relieve destructive emotions, relax the spirit and promote social interaction behavior. Consciously create some popular science education learning environments and game areas, and set up spaces that meet the needs of autistic children for rehabilitation (Kelly, 2018). Ludlow et al. (2020) believes that the education and rehabilitation space for autistic children should meet the essential functions of education and living and the functions of learning, playing and treatment. Consequently, creating a good healing environment for them so that the education and rehabilitation of autistic children can achieve twice the result with half the effort (Ludlow et al., 2020). Klin & Volkmar (2000) believes that reasonable and adequate functional zoning of the teaching and rehabilitation space for autistic children can promote better communication and learning between autistic children and rehabilitation therapists and effectively promote the rehabilitation progress of autistic children.

Currently, the indoor layout of the education and rehabilitation space for autistic children mainly depends on manual work, with a heavy workload and low efficiency. It is of great research value and practical significance to explore the intuitive design and optimization of the spatial layout of education and rehabilitation for autistic children. The intuitive design and optimization of interior space layout can improve the efficiency of design work, reduce the threshold of layout design, and allow users to participate in the internal space layout design process by reducing designers’ interior design work (Zheng et al., 2019). Marson & Musse (2010) proposed a design system that generates a set of configurations according to the given constraints and prioritizes them according to multiple evaluation indicators to find the best functional structure of rooms in the building layout. Chen et al. (2015) proposed a semi-automatic layout modeling system for creating the interior plan of residential buildings and integrating it with existing map data to generate building models. The intelligent indoor space design method realized in this study can meet the real-time layout requirements of the indoor space design platform. The layout recommendation algorithm effectively improves the design efficiency and has good use value and research significance.

## Related works

Many scholars have found that the theory of affordability is of great significance in the application of education and rehabilitation space for autistic children. Yatiman, Aziz & Said (2012) found that it plays a vital role in children’s social interaction and cognitive level development by studying the environmental affordability of children on their way to school. Kyttä (2003) divided affordability into three levels in his research, namely perceived affordability, used affordability and shaped affordability to describe the degree and characteristics of human interaction with the environment. Objects in the environment must have the function of being used if they are to be available. Turner & Turner (2002) believes that human perception is related to human behavior to a certain extent, and the corresponding behavior is generated through the information obtained from vision and perception. Dominguez, Ziviani & Rodger (2006) elaborated on the combination of play therapy and space design, recording play behavior in the clinical play environment and providing a new means for evaluating and treating autistic children. Maule et al. (2018) studied autistic children’s adaptability to color, demonstrated autistic children’s color cognition and preference, and provided evidence for autistic patients’ specific color adaptation. Rogers et al. (2003) discussed the influence of autistic children’s behavioral characteristics on space and found that the overall imitation ability of autistic children is related to visual-spatial skills.

For the research on the indoor space layout of the education and rehabilitation space for autistic children, a popular method is to realize the layout plan design through expert systems. Lin et al. (2015) developed facility layout design models by combining expert systems and artificial intelligence ideas. Sharma & Park (2018) encapsulates the architectural design knowledge into the expert system to evaluate the layout plan of the building according to the relevant design rules and constraints and give reasonable modification opinions. The other method is to use shape syntax to design the layout plan. The shape syntax contains a set of given generative rules. Designers can apply different transformation rules from the initial to the final design (Guo, Zhang & Zhong, 2014). Deng et al. (2020) proposed a series of layout plan generation algorithms based on the improved version of the hybrid evolutionary algorithm, mainly used in the early architectural design stage. Hutter, Hoos & Leyton-Brown (2011) also uses evolutionary algorithms to develop an interactive layout design system, which can generate personalized space layouts according to design criteria and user preferences to assist designers in layout planning. Merrell, Schkufza & Koltun (2010) proposed a data-driven method for generating the layout plan of indoor space. The method uses a Bayesian network to learn the relationship and related attributes between rooms from the house-type diagram’s data set and generate the indoor space layout based on a bubble diagram using a random optimization method. Wei et al. (2018) proposed a data-driven method to estimate the size and direction of the room in the interior form and learned relevant metrics to plan appropriate space layouts. Liu et al. (2013) proposed an interactive layout design method, which combines design constraints, user preferences and manufacturing considerations to optimize spatial structure. Grant & Osanloo (2014) proposed a method to explore different layout design schemes. To obtain the spatial layout with changing connections, the random optimization method is used as the optimization algorithm. Alpert et al. (2014) proposed a method to generate irregular spatial arrangements automatically. Given a pattern template, this method can generate various layouts that meet users’ geometric and topological requirements. Through in-depth learning, Liu et al. (2017) obtains vectorization results from the indoor layout plan represented by a pixel map. Their method mainly uses integer programming to encode design constraints.

However, the layout problem is an NP-hard problem (Puhachov et al., 2021), that is, the layout problem of a complex system cannot determine whether to find a solution in polynomial time, and there is often a combination explosion in the solution scale, which makes it difficult for general optimization methods to find an effective solution in a short time, leading to unsatisfactory optimization results. This research proposes a two-stage deep learning network for indoor space layout design to solve this problem. By building a reasonable model and extracting more expressive features, the efficiency and accuracy of the structured learning algorithm are effectively improved, and better indoor scene layout estimation is achieved. The experimental results show that our method is feasible and effective.

## Evaluation of education and rehabilitation space for autistic children based on affordance theory

**Figure 1:**
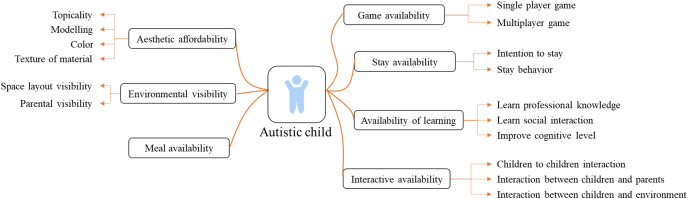
Affordability factors of education and rehabilitation space for autistic children to adapt to children’s characteristics.

This study used affordability as the guiding principle for designing educational rehabilitation spaces for autistic children. We analyzed the affordability of education and rehabilitation space for autistic children, classified and listed the environmental affordability factors according to the characteristics and needs of autistic children, and provided theoretical references for the subsequent research on space design. The affordability factors of education and rehabilitation space for autistic children to adapt to children’s characteristics are shown in Fig. 1.

The education and rehabilitation space for autistic children should consider the factors that children can perceive. Based on the characteristics of autistic children’s perception, spatial behaviour and actual needs, The affordability factors in the education and rehabilitation space for autistic children that can be perceived by children and provide children with corresponding values and meanings can be summarized as affordability of games, interaction, meals, affordability of stay, environmental visibility, aesthetics, learning and safety. The affordability of play refers to the value of play and entertainment that the environment can provide for autistic children, the affordability of interaction refers to the value that the environment can help autistic children enhance social interaction and promote parent-child feelings, the affordability of meals refers to the functional value that the environment can bring to the dining subject, and the affordability of stay refers to the value that the environment can extend the residence time of autistic children and stimulate their various behaviors in space, environmental visibility refers to the value that space gives autistic children a sense of inner security, aesthetic affordability refers to the value that environment can create attraction for autistic children, learning affordability refers to the value that various affordability factors in the environment can play in learning multiple aspects of knowledge for autistic children, and safety affordability refers to the value of space creation to ensure the safety of autistic children’s activities. The analysis of environmental affordability factors for education and rehabilitation space for autistic children is shown in Table 1. Each affordability factor can be subdivided into multiple categories; each category contains numerous affordability components, which provide specific functional indicators for the education and rehabilitation space of autistic children through the combination of environmental factors with different characteristics.

**Table 1:** Analysis of environmental affordability factors for education and rehabilitation space for autistic children.

Affordability	Classification	Features	Affordability component factor	Value and significance
Game affordability	Single player game	Quiet and independent	1. Independent game table and chair combination 2. Division of static independent game function areas	1. Enrich the educational experience of autistic children 2. Improve the fun of the space
	Multiplayer game	Active and cooperative	1. Game facilities for large-scale activities 2. Dynamic multiplayer game area division	
Interactive affordability	With autistic children of the same age	Body interaction and language interaction	1. Focus on the infrastructure of common play for autistic children 2. Division of interactive areas with spatial centripetal force	Promoting social interaction of autistic children
	With parents	Body interaction and language interaction	1. Provide basic furniture for autistic children to interact with parents 2. Comparison of furniture scales adapted to the two groups	Promote parent-child communication and interaction, and enhance parent-child relationship
	With the environment	Physical and visual interaction	1. Interactive devices in the environment 2. Interaction between space interface modeling and people	Improve the attraction of space environment to people and enrich space experience
Meal affordability	Dining with parents	Behavior restraint and quiet dining	1. Dining furniture combination 2. Dining area division 3. Functions of different types of space areas	Meet the basic function orientation of the theme restaurant
Stay affordability	Intention to stay	External environment intervention	1. Surface modeling pattern design that attracts users to stay 2. Possible functional area division for multiple behaviors 3. Furniture facilities with rest function	Provide a relaxed and pleasant environment for users
	Stay behavior	Subjective will guidance of users	Attraction is guided by the material characteristics observed by vision, and the corresponding willingness to stay is generated to guide the subject to form retention behavior	Extend the space retention time of autistic children, and provide possibility for deeper parent-child communication and social interaction of autistic children
Environmental visibility	Visibility of layout design around the space	Observable behavior	1. The space layout with open and closed characteristics is mainly characterized by open and semi open spaces 2. Furniture and surface shape design with specific functional attributes	Promote autistic children’s understanding of spatial layout and stimulate their desire for spatial exploration
	Visibility to accompanying parents	The wait-and-see behavior of gaining inner security	Layout of parents’ rest area accessible to autistic children	Meet the psychological needs of autistic children’s sense of security during independent activities, and ensure the observability of autistic children’s activities
Aesthetic affordability	Aesthetic modelling	Through visual perception and forming the overall impression of space	Surface form, functional layout, light environment, physical display	Promote the aesthetic level of autistic children and improve their aesthetic perception
	Color matching aesthetics			
	Theme relevance aesthetics			
Affordability of learning	Learn professional knowledge	Promote the development of the cognitive level in the subconscious through only perception	Learning area, learning appliance, interface with spatial centripetal force	Improve the cognitive level of autistic children
	Improve cognitive level			
	Learn social interaction			

As for the available content of the education and rehabilitation space for autistic children that children and their parents can perceive, the functional layout of the teaching and rehabilitation space for autistic children must consider the available factors for the two groups simultaneously. Therefore, the functional structure of the education and rehabilitation space for autistic children must have three content areas: interactive area, children’s game area, and parents’ waiting area. This study refines the specific functional layout of these three regions based on the actual situation of the education and rehabilitation space for autistic children.

## Design of education and rehabilitation space for autistic children based on bidirectional lstm and word embedding algorithm

This research proposes a layout recommendation algorithm based on deep learning, which is used to improve the layout efficiency of the education and rehabilitation space for autistic children and realize real-time online layout. The scene information is digitized in binary code. The segmentation and layout network models are constructed through bidirectional LSTM to discover the long segment pre-segmentation of house type and obtain the layout results. The word embedding algorithm is used to abstract the cross features between each vector segment, and the dimension of the feature matrix is reduced to improve the speed and accuracy of the layout scheme recommendation.

### Scene information extraction

**Figure 2:**
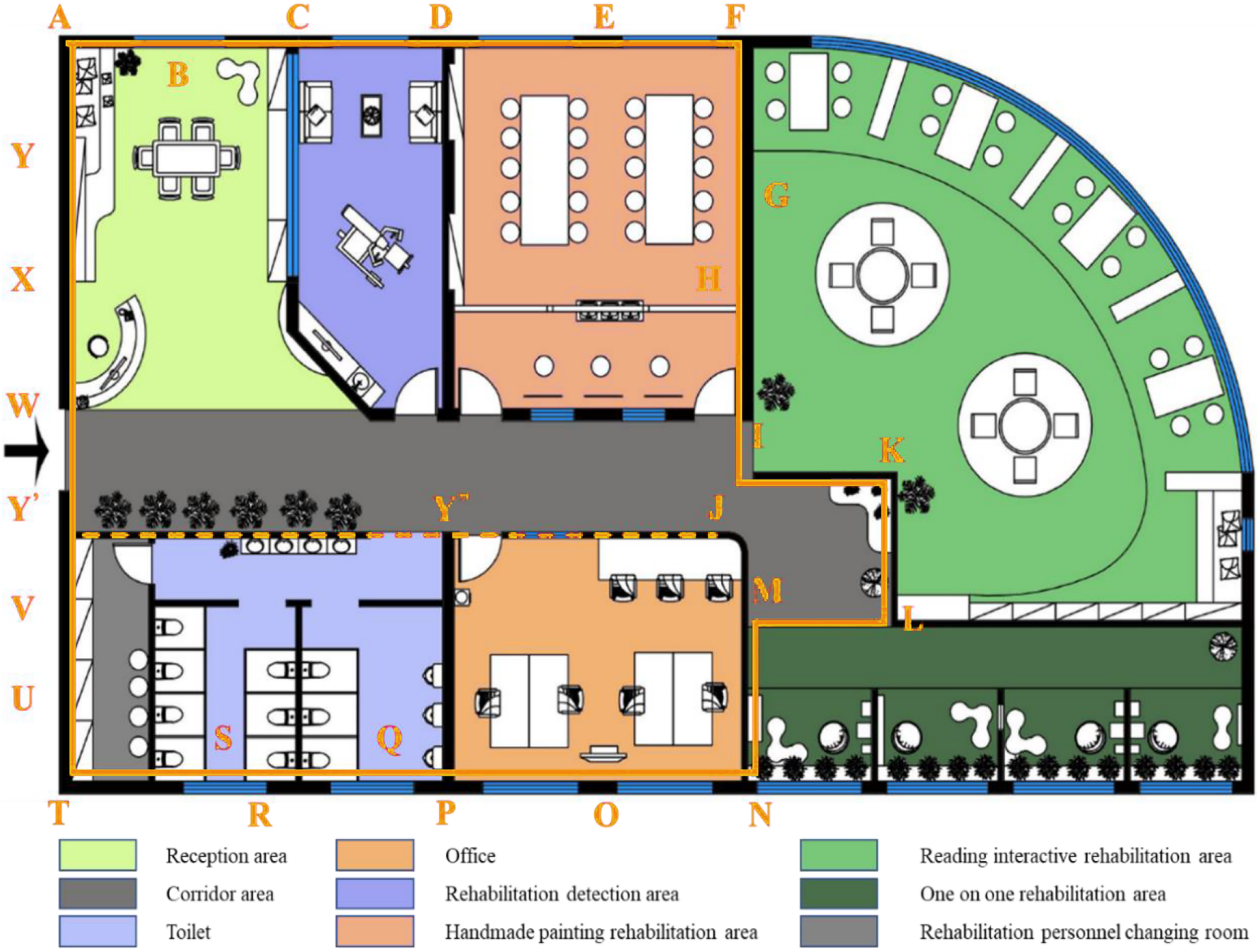
Layout plan of education and rehabilitation space for autistic children. Figure 2 is a top view of the house-type space that has completed the education and rehabilitation of autistic children.

A house-type diagram is a meaningful way to describe indoor scene information. Figure 2 is a top view of the house-type space that has completed the education and rehabilitation of autistic children. Considering the planarity of layout problems in interior design, this study uses the layout information of a two-dimensional plan to describe the layout problem of three-dimensional space, and the problem of spatial layout scheme recommendation is converted into the issue of planar layout scheme recommendation.

The house-type information extraction is to express the house-type information efficiently and accurately. In this study, the house type segment is used as the basic unit to describe the house type information in the house type map. The natural envelope line is the area to be laid out for the guest restaurant. House-type segment refers to a vector segment such as $\overrightarrow {{\rm BC},} \overrightarrow {{\rm CD}} ,\overrightarrow {{\rm DE}} ,\overrightarrow {{\rm EF}}$, which is separated by endpoints with different inherent properties of house type. Thus, the area to be laid out in the figure can be described by house-type segment sequence $\left( {\overrightarrow {{\rm AB},} \overrightarrow {{\rm BC},} \overrightarrow {{\rm CD}} ,\overrightarrow {{\rm DE}} , \ldots ,\overrightarrow {{\rm WX},} \overrightarrow {{\rm XA}} } \right)$. Each segment corresponds to a layout label. For example, $\overrightarrow {{\rm BC},} \overrightarrow {{\rm DE}}$ corresponds to an ordinary wall, $\overrightarrow {{\rm CD}}$ corresponds to a balcony door, and $\overrightarrow {{\rm EF}}$ corresponds to a TV cabinet combination. Similarly, the layout result of the area to be layout can be represented by the layout tag sequence, and Table 2 is the tag value corresponding to the layout classification result.

**Table 2:** Layout classification results.

Ordinary wall	TV cabinet combination	Sofa combination	Table	Shoe cabinet	Window	Balcony door	Entrance doors
0	1	2	3	4	5	6	7

In Table 3, it is agreed to use 12-bit binary codes of three fields to represent the characteristics of a house-type vector segment. The first field represents the properties of the house type segment, including ordinary walls, windows, bedroom doors, *etc*. The second field represents the direction of the vector segment. The approach here is relative. That is, the direction of the house-type vector segment is different when the starting point and direction of the coding are different. If point B is used as the starting point for counterclockwise encoding, the binary encoding of the direction value of segment BC is 10, while the direction value is 00 for clockwise encoding; The third field stores the length characteristics of the house type segment, with an error of ±0.5 dm. According to the layout principle of the interior design industry, this error range does not affect the result of the layout. For example, for a house-type segment with a standard wall attribute, direction value of 01, and length of 4 m, the binary code value is 000010101000, which can be represented by decimal 168. Thus, the features of each house-type vector segment can be represented by a decimal number. The number of house-type segment features is 212, and the range of encoding values is [04095].

**Table 3:** Feature code of house type section.

Attribute								Direction				Length			
111	110	101	100	011	010	001	000	11	10	111 111	…	000 001	…	0000 001	000 000
Other	Entrance doors	Kitchen door	Balcony door	Toilet door	Bedroom door	Window	Ordinary wall	↓	←	↑	→	127	…	1	0

According to the layout principle in interior design, the house section with a length of more than 4 m may be divided into multiple areas for layout. The endpoint of the inherent property of the house type cannot directly separate this long segment. It is necessary to consider the spatial structure characteristics of the overall house type and then screen out the correct segmentation point. This research combines the rules of interior design and pre-segments the long section of the house type through such segmentation candidate points as L-shaped wall corner projection point, I-shaped wall endpoint projection point, I-shaped wall midpoint projection point, T-shaped wall intersection projection point, T-shaped wall intersection point, *etc*. The real segmentation point will be selected from these segmentation candidate points. After the long segment is correctly pre-segmented, the long segment’s information is described as a segmentation candidate segment sequence.

**Figure 3:**
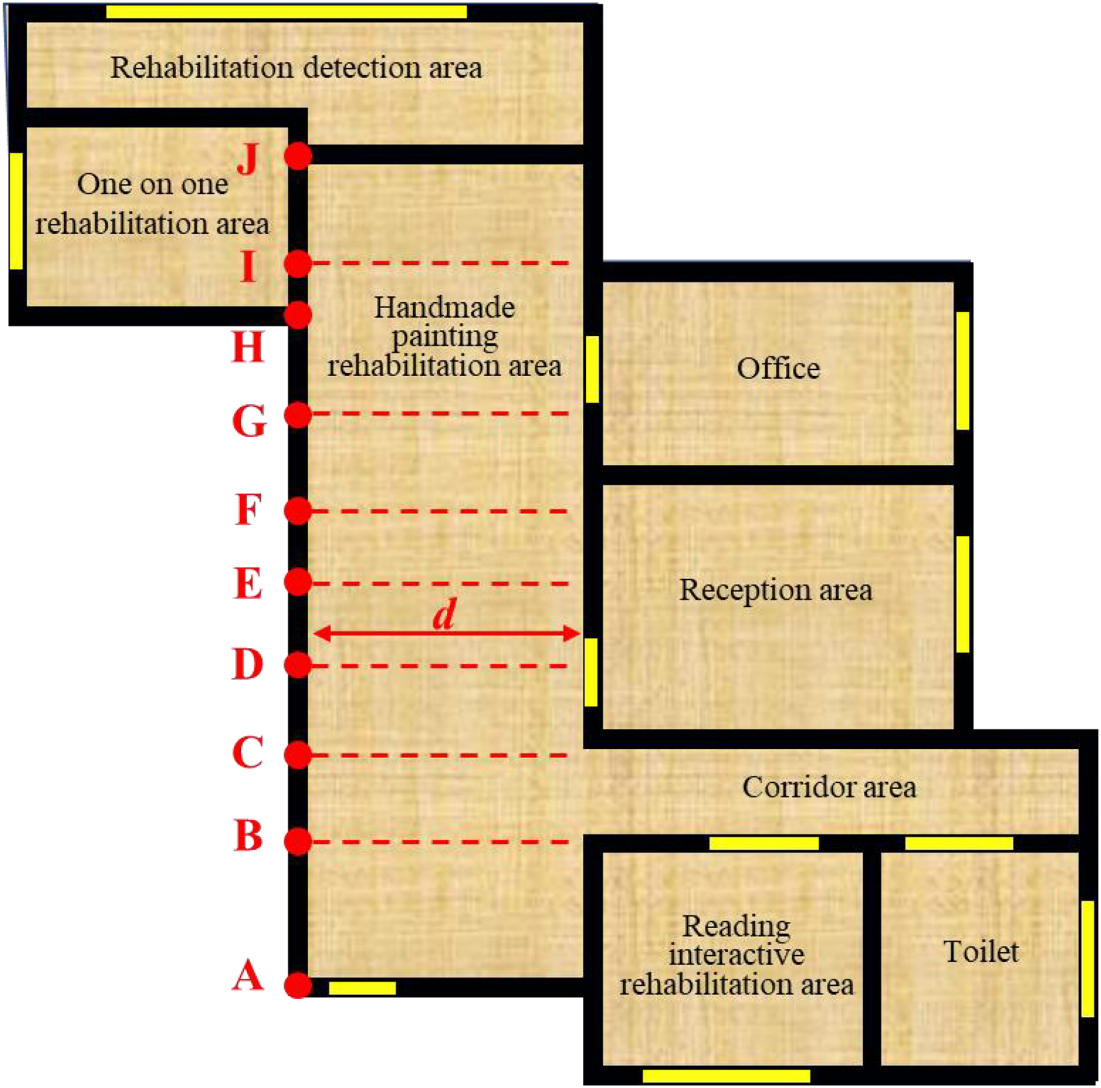
Schematic diagram of segmentation candidate points.

Similarly, the classification result of the segmented segment can be represented by the segmentation result tag sequence. Again, three fields with a total of 16 bits of binary code are used to characterize the characteristics of the segmentation candidate segment, and the number of features is 216. The first field represents the attribute of the endpoint of the split segment. Since each element has two endpoints, the attribute of the endpoint direction of each selected component is used as the attribute of the candidate segment according to the coding law of the split candidate segment. To unify the number of split candidate segments and candidate points, here is the last element in the sequence plus the attribute “segment endpoint”. It is agreed that the “segment endpoint” must be the split point. For the long section $\overrightarrow {{\rm AJ}}$ in the dining room area in Fig. 3, with A as the coding starting point, B and C are the projection points of the L-shaped wall corner, D and G are the projection points of the endpoints of the straight wall, E as the projection point of the middle point of the straight wall, F and I are the projection points of the intersection of the T-shaped wall, H is the intersection of the T-shaped wall, and J is the end point of the section; The second field stores the length characteristics of the segmentation candidate segment; The third field is segment distance, which refers to the distance between the midpoint of the segmentation candidate segment and the opposite segment in the layout space, fully expressing the space size of the segmentation candidate region. Figure 3 shows the segment distance of the segmentation candidate segment $\overrightarrow {{\rm DE}}$.

For the long segment $\overrightarrow {{\rm AB}}$ in Fig. 3, the segmentation candidate points are the projection points ð′ and ð′′ of inflection points S and F, then the long segment $\left( {\overrightarrow {{\rm A}{{\rm Y}^{\rm '}},} \overrightarrow {{\rm {Y}^{\prime}{Y}^{\prime \prime}}} ,\overrightarrow {{\rm {Y}^{\prime \prime}B}}}\right)$ is described by dividing the candidate segment sequence C. The two segmentation candidate points here are valid segmentation points, so the corresponding classification result of the segmentation segment is (1, 1, 1). After segmentation preprocessing, the area to be laid out is fully described in the form of a house-type segment sequence $\left( {\overrightarrow {{\rm A}{{\rm Y}^{\rm '}},} \overrightarrow {{\rm {Y}^{\prime}{Y}^{\prime \prime}}} ,\overrightarrow {{\rm {Y}^{\prime \prime}B}} ,\overrightarrow {{\rm BC}} ,\overrightarrow {{\rm CD}} ,\overrightarrow {{\rm DE}} \ldots ,\overrightarrow {{\rm WX}} ,\overrightarrow {{\rm XA}} } \right)$. The label of the corresponding layout scheme is $(3,0,2,0,6,0\dots7,0)$.

In this study, the layout scheme data is processed in the above way. So far, the recommendation problem of the layout scheme has been transformed into the classification problem of segmentation and house type segments. Next, we will build a segmentation segment classifier and house type segment classifier, respectively, through a neural network model to realize the automatic layout of the space to be arranged.

### Construction of network model

The layout scheme recommendation model in this study includes two parts: the segmentation network model and the layout network model. Because the description form of the segmentation candidate segment sequence is similar to that of the house segment sequence, the house segment sequence is taken as an example to describe the process of matrix transformation of sequence data and matrix dimension reduction. The above digitizes the house-type segment information, and its feature number is 4,096—some house-type segment codes may be 1, 2, or 4,000. The description of the distance between house-type segments is unreasonable. In addition, when classifying house-type components, the coding values of house-type elements are different, but the classification labels may be the same. Therefore, further sequence data processing is required to make the distance between house-type vector segments more reasonable.

A standard method is to use one-pot coding. It is to create a feature library, number each feature sequentially from 0 to 4,095, and map each code value to a 4,096-dimensional vector. The code value can be mapped to a vector $(0, 0\ldots 0, 1, 0, 0\ldots 0)$ where only the i+1 column is one and the other columns are 0. Figure 3 shows the process of obtaining the characteristic matrix by one pot coding from the house type segment sequence. The essence of one-pot coding is to normalize the distance between features, and each element is independent of the other. For the data of the house type segment and segmentation segment studied in this study, this approach ignores the similarity and correlation between coding components. Moreover, its dimension is too high after the sequence is matrixed, especially for segmentation segments. After one pot coding, the dimension is up to 60,000, which also challenges the calculation of the layout recommendation model.

### Word embedding algorithm

**Figure 4:**
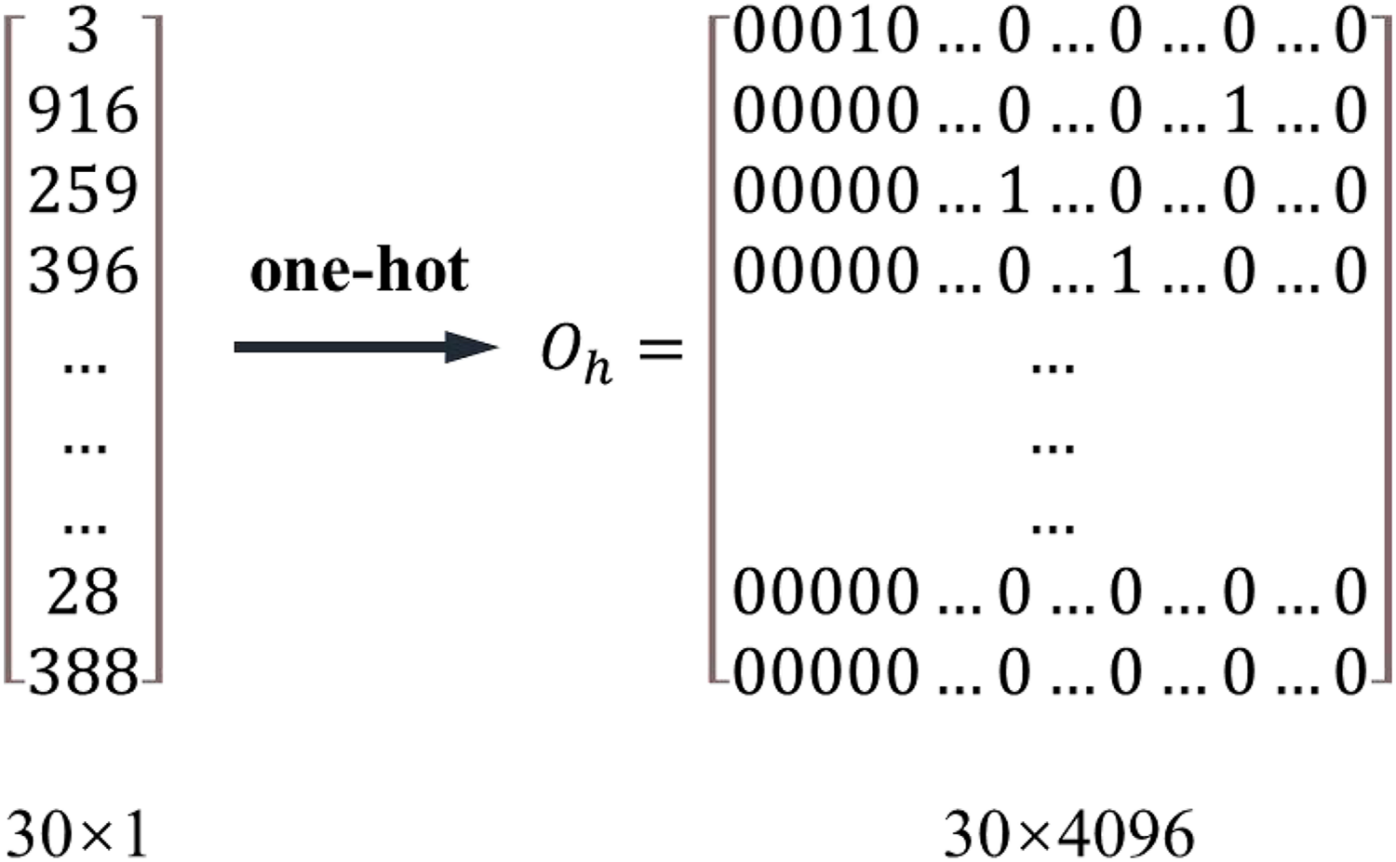
One-hot coding.

To make the distance between vectors more reasonable and reduce the dimension of the feature matrix, this study uses a word embedding algorithm to process the digitized house-type information. The word embedding algorithm converts positive integers into floating point dense vectors of fixed size. Take the sequence of house-type segments in Fig. 3 as an example. The number of features of the house type segment is 4,096. The feature matrix ${{\rm O}_{\rm h}}$ is obtained by one-hot coding, and each feature is independent of the other; The word feature matrix ${{\rm M}_{4096,64}}$. Figure 4 is used to obtain the distributed representation of terms, which realizes the abstract extraction of features and the expression of the potential relationship between coding values. The first line of the word feature matrix represents the embedding of the word vector with the first item in the one-pot code, and the word embedding of words will be continuously optimized with the model’s training. Equation (1) is the dimensionality reduction calculation method of the feature matrix, where ${{\rm M}_{{\rm n},{\rm k}}}$ is the feature matrix, ${{\rm O}_{\rm h}}$ is the feature matrix obtained by one-hot coding, and ${\rm M}_{{\rm m},{\rm k}}^{{\rm word\; vector}}$ is the dense feature matrix reduced by the word embedding algorithm. After dimension reduction, the 4,096-dimension vector in Fig. 4 is reduced to 64 dimensions.

(1) $${\rm M}_{{\rm m},{\rm k}}^{{\rm word\; vector}} = {{\rm O}_{\rm h}}{{\rm M}_{{\rm n},{\rm k}}}$$

**Figure 5:**
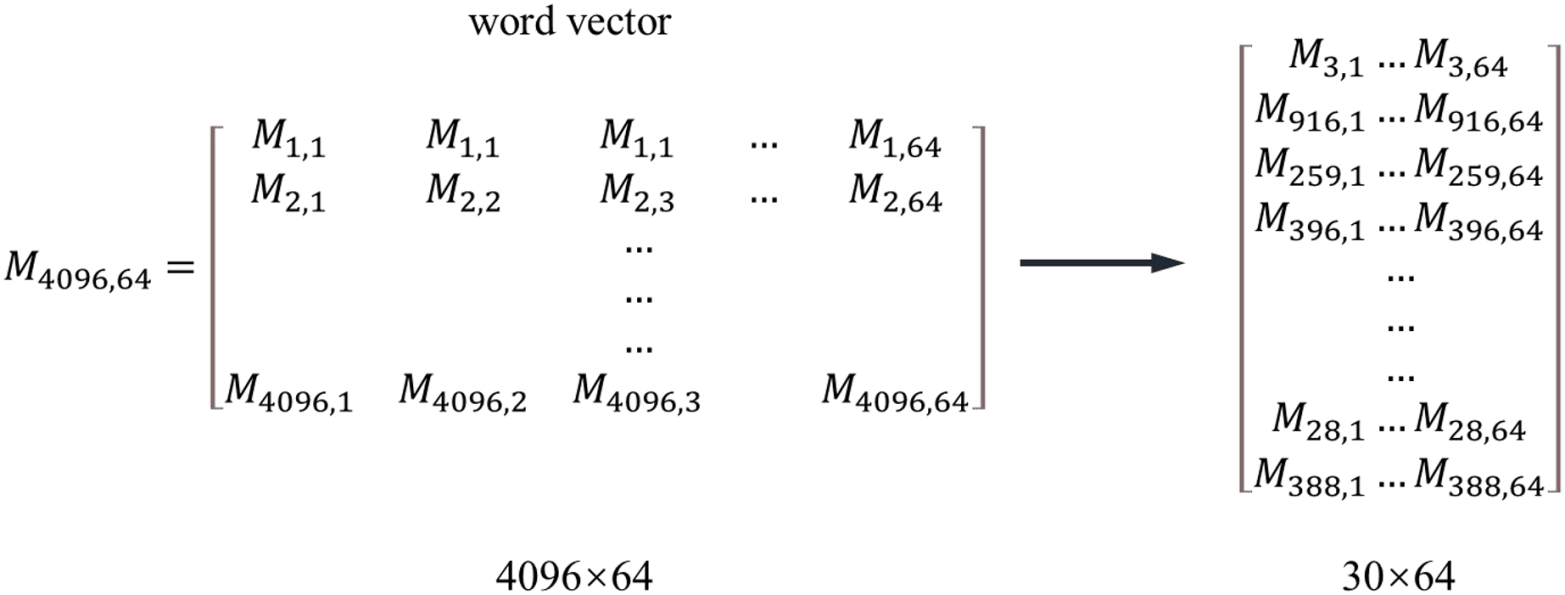
Embedded word representation.

In the collocation recommendation model of this study, the PCA algorithm is used to reduce the dimension of the image feature matrix. Since the goal of PCA is to extract the most valuable information based on variance, the data labels are meaningless after dimension reduction, so it is more suitable for unsupervised learning and privacy information processing. Unsupervised learning generally uses clustering to classify samples. Supervised learning backpropagates through the error between the actual output and the predicted value to modify the weights and complete the network modification. The segmentation and house-type segment classifiers studied in this chapter belong to supervised learning. The word embedding algorithm can reflect the cross features between segments, and the feature distance between each component constructed by it is more reasonable. The embedded word representation is shown in Fig. 5.

**Figure 6:**
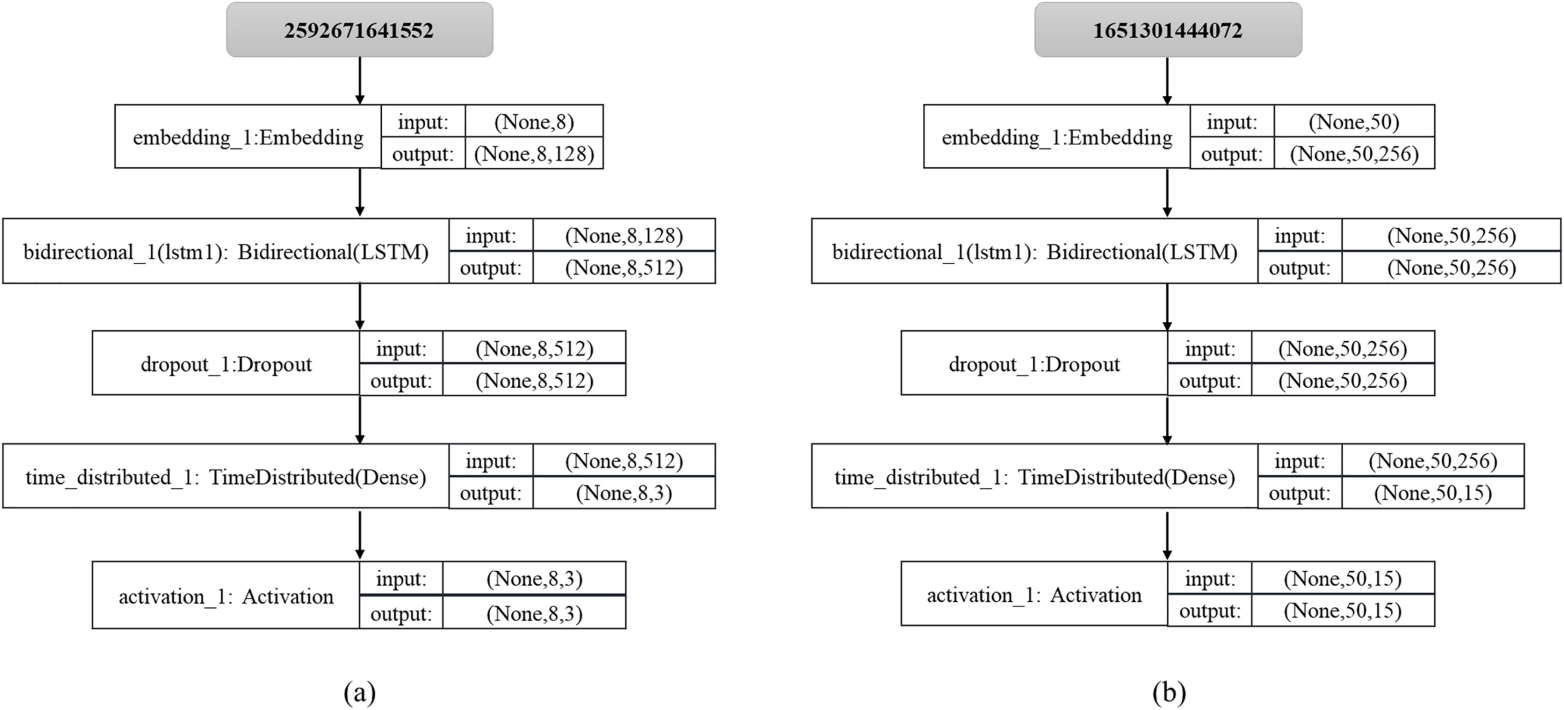
(A) Split network model; (B) layout network model.

The classification results of this study’s house type and segmentation segment classifier are related to the first half of the sequence and closely associated with the second half of the sequence. Therefore, this study uses a two-way LSTM model to implement a layout recommendation algorithm. The segmentation network model is shown in Figs. 6A and 6B is the layout network model. In this study, dropout is used to randomly drop some network nodes and not participate in the calculation of forward transmission, to reduce the complexity of the model and alleviate the overfitting.

### Layout recommendation algorithm flow

To sum up, this study implements the recommended algorithm of layout scheme according to the following process:

(1) First, according to the input house type scene information, the house type segment sequence $\left(\overrightarrow {{{\rm w}_1}} ,\overrightarrow {{{\rm w}_2}} , \ldots ,\overrightarrow {{{\rm w}_{\rm i}}} , \ldots ,\overrightarrow {{{\rm w}_{{\rm n}}}}\right)$ Describing the space to be laid out is preliminarily constructed. The attributes, direction, length, and other characteristics of each house type segment B are embedded into the binary code to digitize the scene information.

(2) If $\left| {\overrightarrow {{{\rm w}_{\rm i}}} } \right| > 4\;{\rm m}$ exists, and there is a segmentation candidate point ${{\rm C}_1},{{\rm C}_2},{{\rm C}_3} \ldots {{\rm C}_{\rm m}}$ on the segment, use the candidate segment sequence $\left( {\overrightarrow {{{\rm c}_1}} ,\overrightarrow {{{\rm c}_2}} \ldots \overrightarrow {{{\rm c}_{\rm i}}} \ldots \overrightarrow {{{\rm c}_{\rm m}}} } \right)$. After pre-segmentation of segmentation, the candidate point describes the long segment to be segmented and embeds the segmentation point attribute, segment length, segment spacing and other features of each candidate segment $\overrightarrow {{{\rm c}_{\rm i}}}$ into the binary code to digitize the information of the segment to be segmented. The correct segmentation points can be identified through the trained segmentation network model. The segmented house segment sequence is $(\overrightarrow {{{\rm w}_1}} ,\overrightarrow {{{\rm w}_2}} \ldots \overrightarrow {{{\rm c}_1}} \ldots \overrightarrow {{{\rm c}_{\rm m}}} \ldots \overrightarrow {{{\rm w}_{{\rm n})}}}$, which can completely describe the information of the space to be arranged.

Otherwise, no segment in the sequence needs to be split, so execute (3) directly.

(3) The trained layout classifier is used to classify the processed house segment sequence, and the layout label sequence is obtained.

(4) Place the home object model according to the layout result in (3). In this study, scene information is derived from the guest restaurant scene well arranged on the interior space design software platform. After data expansion operations such as rotating, mirroring, and changing the starting point of coding for the house type scene, 10,000 labeled segmentation long segment data and 30,000 labeled household type scene to be arranged are used as data sets to train the segmentation network model and layout network model respectively, of which the training set The ratio of verification set to test set is 8:1:1. What needs to be added here is that the data set used for the layout network model contains the layout data after accurately segmenting long segments. The layout result is considered inaccurate if the long pieces in the current house-type space to be distributed are not accurately segmented.

## Results and discussions

### Word embedding vector dimension OUTPUT_DIM

**Figure 7:**
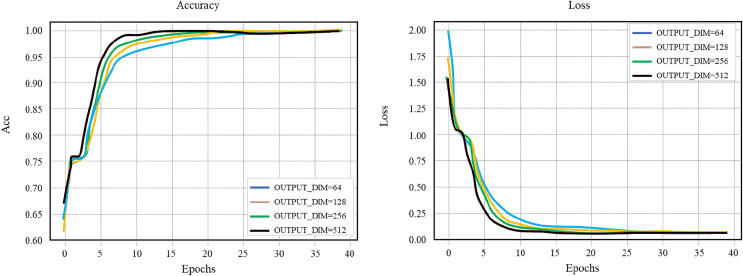
Layout network model comparison.

Using different OUTPUT_DIM values for comparison experiments, we get the accuracy curve and loss value curve of the training set of the layout network model, as shown in Fig. 7. Set the learning rate to 0.1, the number of hidden layer nodes HIDDEN_SIZE to 128, and the batch to 256. When the OUTPUT_DIM is 256 or 512 and the round training Epochs reaches 20, the training curve reaches saturation and the optimal accuracy reaches 100%. To reduce the complexity of the model and speed up the training, the OUTPUT_ DIM of 256 is selected in this study to continue the comparative follow-up experiment.

### Number of hidden layer nodes HIDDEN_SIZE

**Figure 8:**
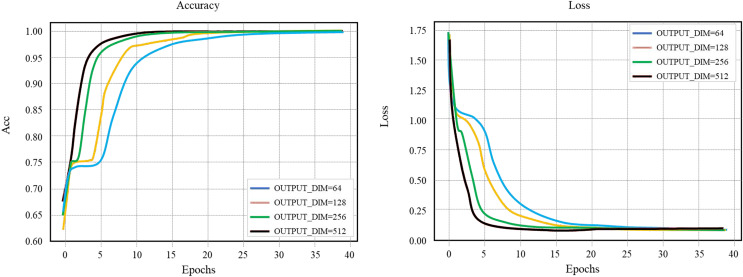
Layout network model comparison.

The comparison experiment diagram of the layout network model HIDDEN_SIZE is shown in Fig. 8. The selected learning rate is 0.1, the number of hidden layer nodes. OUTPUT_DIM is 256, the batch_size is 256, and the selected HIDDEN_SIZE is 128.

### Selection of data batch_size

**Figure 9:**
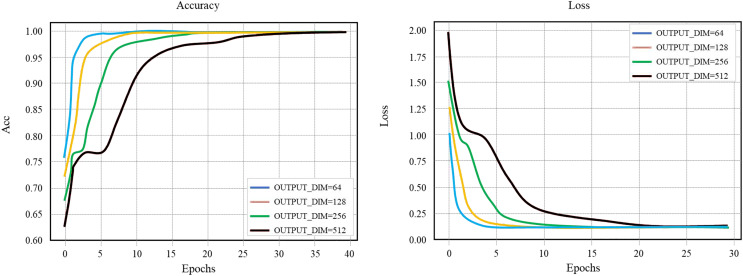
Comparison of the batch_size of the layout network model.

In the model training stage, we used different batch size values to conduct comparison experiments and obtained the curve of the training set accuracy and loss *vs*. the number of iterations, as shown in Fig. 9. According to the curve, the best accuracy can reach 100%. Because the size of the batch size will affect the training speed, we comprehensively consider the smoothness of the curve and the training speed and select the size of the batch size as 128.

### Selection of learning rate

**Figure 10:**
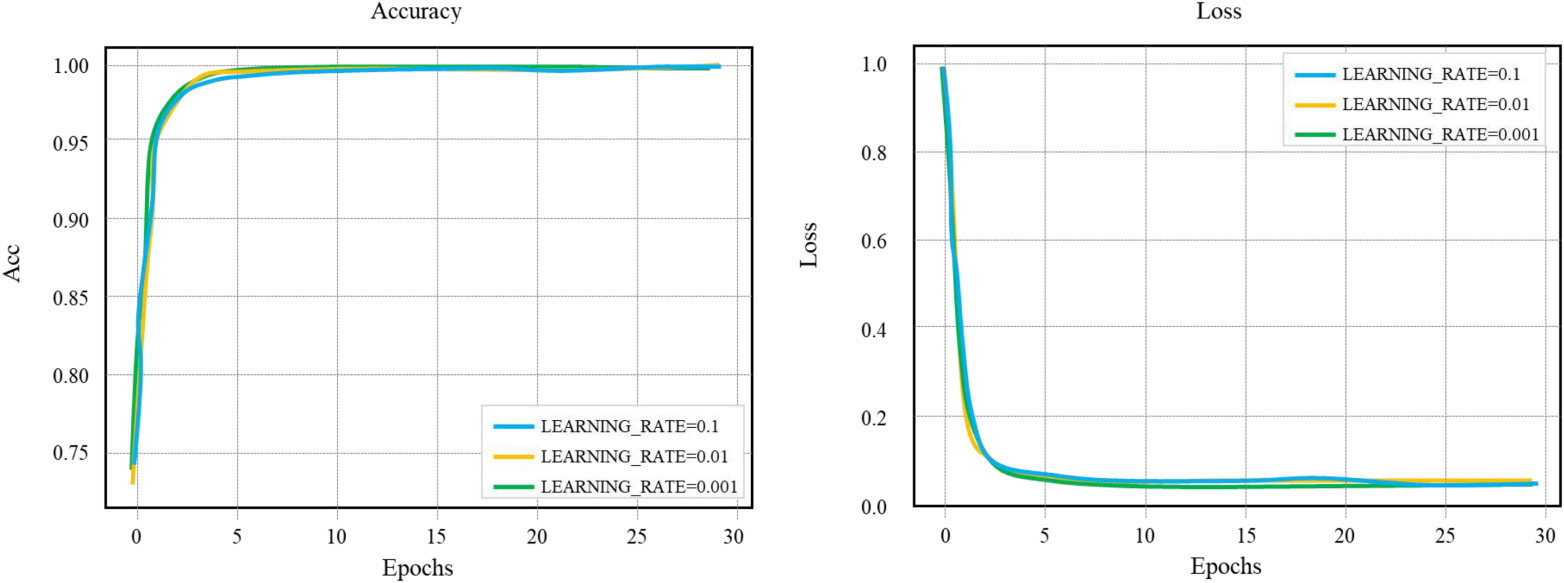
Comparison of LEARNING_RATE of layout network model.

The learning rate determines whether the objective function can converge to the local minimum and when it will converge to the minimum. When the learning rate is set too small, the convergence process will be prolonged; when the learning rate is set too high, the gradient may oscillate around the minimum value or even not converge. Control other parameter variables. Figure 10 is a comparative experiment diagram of LEARNING_RATE of the layout network model, and the learning rate is selected as 0.1.

**Figure 11:**
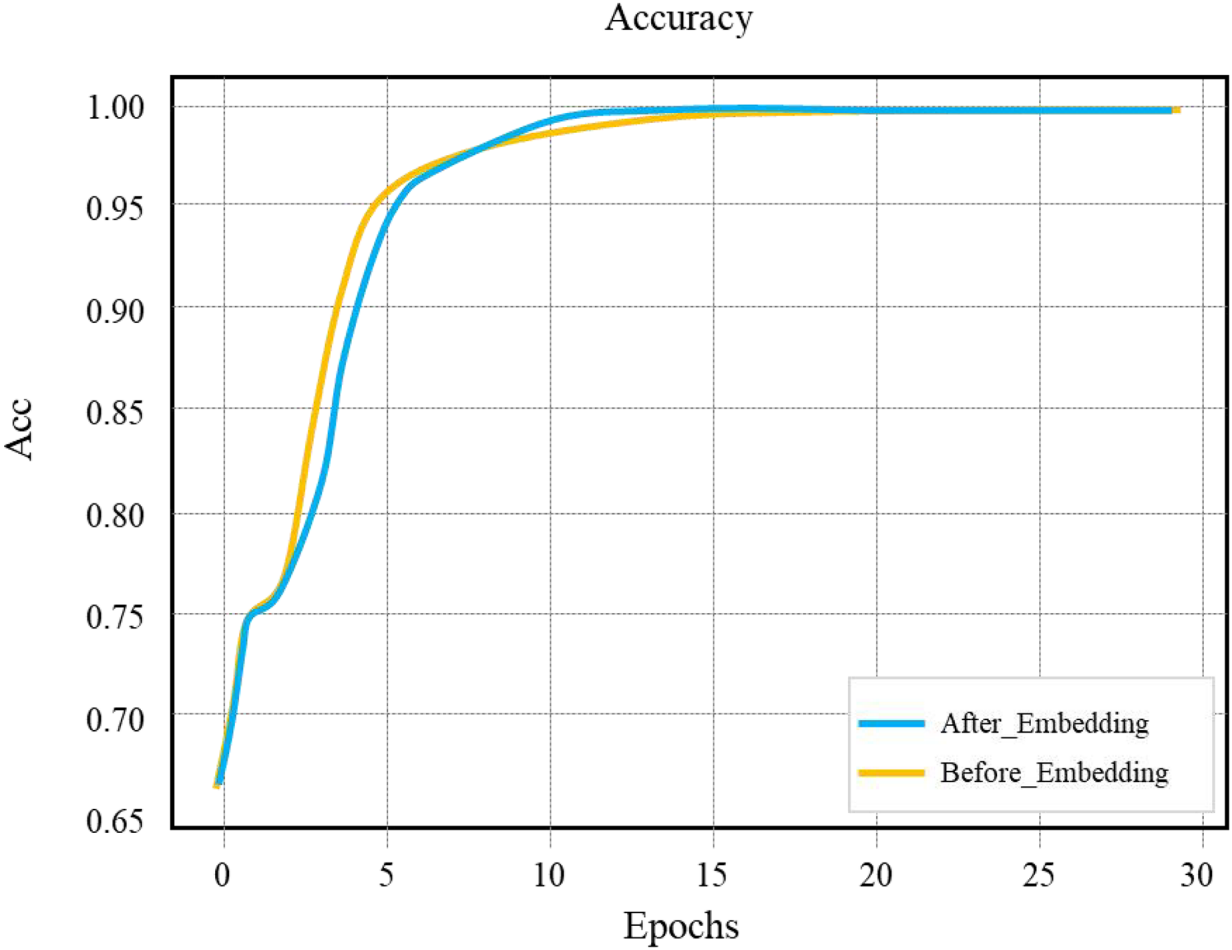
Comparison of embedding accuracy of layout network model training set.

According to the layout network parameters determined above, we choose 256 for OUTPUT_DIM, 128 for HIDDEN_SIZE, and batch_size 128, and the learning rate LEARNING is 0.1. The final network model training set accuracy curve is drawn as “After_Embedding” in Fig. 11. The “Before_Embedding” curve in Fig. 11 is the training result of the network model without adding the word embedding layer. The model training effect without adding the word embedding layer is better in the early training stage. In the late training stage, the model training effect with adding the word embedding layer is slightly better, and the absolute optimal training accuracy is close to 1. The training duration of the “Before_Embedding” network model is 104 min, and the “After_Embedding” network model is 26 min, which shows the dense processing of the Embedding layer can significantly speed up the training of the network model.

For the whole house-type segment sequence, if one of the house-type segments is incorrectly predicted, the prediction result of the house-type segment sequence is considered to be inaccurate, and the value is 0. Taking four times of experimental data, it is calculated that the average accuracy of a single house segment of the layout model without adding the word embedding layer is 89.75%, and the average accuracy of the whole house segment sequence is 73.48%; The average accuracy of a single house segment of the layout model with the word embedding layer added is 98.60%, and the average accuracy of the whole house segment sequence is 92.16%. The average test speed of the After_Embedding network model is 17.88 s/3,000 data, and the average test speed of the Before_Embedding network model is 309.43 s/3,000 data.

By analyzing the above data, we can draw the following conclusions: the layout network model with word embedding layer can better learn the characteristics between scene information and layout, and its performance in the test set is significantly improved compared with the Before_Embedding model; Under the same layout network model, when comparing the average prediction accuracy of a single house segment with that of the whole house segment, the accuracy of the entire house segment of After_Embedding model is 6.53% lower than that of a single house segment, while the accuracy of the house as the whole segment of Before_Embedding model is 18.13% lower than that of a single house segment.

**Figure 12:**
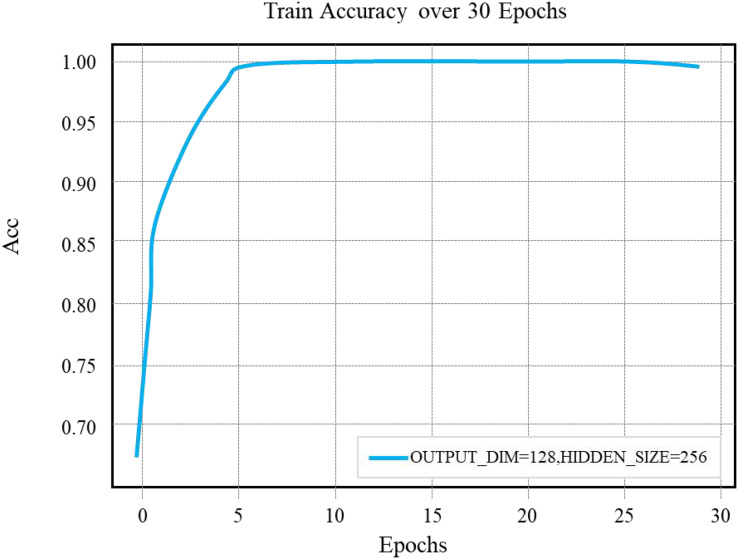
Accuracy of training set of segmented network model.

The accuracy curve of the split network model training set is shown in Fig. 12. The selection process of super parameters is similar to that of the layout network model, so we will not repeat it here. Set the learning rate to 0.01, select the appropriate number of hidden layer nodes HIDDEN_SIZE and word embedding dimension OUTPUT_DIM, and the curve tends to be stable after multiple iterations of training.

This experiment uses 1,000 segmented data to test the accuracy of the segmented network model. The accuracy of the test set is shown in Table 4. The average segmentation accuracy can reach 99.12%.

**Table 4:** Accuracy of split network model test set.

Test set accuracy (%)	1st time	2nd time	3rd time	4th time	Average accuracy
Split network model	99.24	99.01	98.89	99.33	99.12

## Conclusions

When building the scene layout recommendation model, this study incorporates a word embedding algorithm into the neural network model to reduce the extent of a high-dimensional sparse matrix and make the distance between dense features more reasonable, improving the recommendation’s accuracy and speed. The layout network model with word embedding layer can better learn the characteristics between scene information and layout, and its performance in the test set is significantly improved compared with the Before_Embedding model; the accuracy of the whole house segment of After_Embedding model is 6.53% lower than that of a single house segment, and the best accuracy can reach 100%. From the perspective of specific application scenarios, the designed education and rehabilitation space for autistic children meets the essential functions of education and life and can create a good rehabilitation environment for autistic children. There are still some deficiencies in this study and further research is needed. For the inclined wall, dividing the direction range according to the internal design rules and increasing the direction coding is necessary. In the future, we will take this as the starting point of algorithm improvement for research.

## Supplemental Information

10.7717/peerj-cs.1303/supp-1 Supplemental Information 1 Code and data sets.

## Additional Information and Declarations

### Data Availability

The following information was supplied regarding data availability: The data and code are available in the Supplemental File. The data was taken from 3D-FRONT: 3D Furnished Rooms with layOuts and semaNTics: https://tianchi.aliyun.com/specials/promotion/alibaba-3d-scene-dataset.
